# Chunk boundaries disrupt dependency processing in an AG: Reconciling incremental processing and discrete sampling

**DOI:** 10.1371/journal.pone.0305333

**Published:** 2024-06-18

**Authors:** Chia-Wen Lo, Lars Meyer

**Affiliations:** 1 Research Group Language Cycles, Max Planck Institute for Human Cognitive and Brain Sciences, Leipzig, Germany; 2 University Clinic Münster, Münster, Germany; Industrial University of Ho Chi Minh City, VIET NAM

## Abstract

Language is rooted in our ability to compose: We link words together, fusing their meanings. Links are not limited to neighboring words but often span intervening words. The ability to process these non-adjacent dependencies (NADs) conflicts with the brain’s sampling of speech: We consume speech in chunks that are limited in time, containing only a limited number of words. It is unknown how we link words together that belong to separate chunks. Here, we report that we cannot—at least not so well. In our electroencephalography (EEG) study, 37 human listeners learned chunks and dependencies from an artificial grammar (AG) composed of syllables. Multi-syllable chunks to be learned were equal-sized, allowing us to employ a frequency-tagging approach. On top of chunks, syllable streams contained NADs that were either confined to a single chunk or crossed a chunk boundary. Frequency analyses of the EEG revealed a spectral peak at the chunk rate, showing that participants learned the chunks. NADs that cross boundaries were associated with smaller electrophysiological responses than within-chunk NADs. This shows that NADs are processed readily when they are confined to the same chunk, but not as well when crossing a chunk boundary. Our findings help to reconcile the classical notion that language is processed incrementally with recent evidence for discrete perceptual sampling of speech. This has implications for language acquisition and processing as well as for the general view of syntax in human language.

## Introduction

Language allows us to generate and process a huge, possibly unbounded number of word combinations. Not only can we link neighboring words, but also words that are separated by intervening words. Such non-adjacent dependencies (NADs) are attested across languages. In cognitive science, NAD processing is considered to be a working memory task: The first element of an NAD must be memorized until the second element arrives [1–4]. For example, in the sentence *John saw himself.*, *John* must be held until the pronoun *himself*, which refers back to him. Behavioral and electrophysiological studies have shown that infants and adults learn NADs from statistical regularities—our brains monitor the co-occurrence of the first and second elements of NADs [5–11]. The tracking of all possible statistical regularities and the potential grouping of words may be affected by various cues such as transitional probabilities between elements [10, 12–16], prosodic properties of speech [17–20], distributional properties of elements [21–24], and other cues such as function words or morphology for constructing well-formed dependencies [18, 24, 25]. Both adults and infants can learn short NADs in the form of “AXB”, which forms an arbitrary dependency between A and B, interrupted by X (e.g. [7, 10, 23]). While the length of NADs in real languages is not limited to such a short dependency, it is widely accepted that intervening elements disrupt NAD processing. For example, Bock and Miller [26] found that the intervening noun phrase between subject and verb dependency can lead to more errors in a production task that adults tend to produce errors such as *The bridge to the islands were crowded. Corpus analyses revealed that the dependency lengths are shorter than the random baseline across 37 languages [27] and a preference for minimization of the distance between two related syntactic elements was observed [28], suggesting that people have a strong bias toward short dependencies (see also [29]).

**Fig 1:**
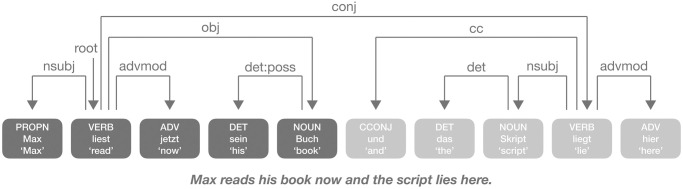
A German example sentence annotated with dependency grammar and chunks. Words with deep and light gray indicate different chunks. Most dependencies form locally within a chunk.

Our ability to learn and process NADs conflicts with evidence that our brain samples speech in larger chunks that contain only a limited number of words. Thus, in principle, chunk endings may be cutting into NADs, as exemplified by Fig 1. In an earlier study, [30] presented long sentences with numerous complex NADs (e.g. *boys who chase dogs see girls.*) to a connectionist network model. The model failed to capture the NADs when the whole sentence was presented at one time. However, when the sentences were presented within a limited processing window of 3-4 words or so chunk-by-chunk, the NAD patterns can be successfully captured by the model. Human chunk-by-chunk sampling is thought to arise from memory limitations: To counteract decay in working memory, we integrate information within limited time windows of up to 3 seconds [31–33]. For language specifically, memory may be restricted to about 2 seconds when articulating a word sequence [34]. In psycholinguistics, a processing time window of six words has been proposed [35], roughly equaling 2.5 seconds when assuming a speech rate of 150 words per minute [36]. Current neuroscientific work suggests that such temporal limitations may arise from a neurobiological constraint: the wavelength of slow-frequency neural oscillations. For instance, a recent study found electrophysiological activity in the delta band (< 4 Hz) to align to phrases and sentences [37]. Specifically, phase angles of delta-band activity predict the boundaries of multi-word chunks [38], particularly when these exhibit a duration of 2.7 seconds [39].

**Fig 2:**
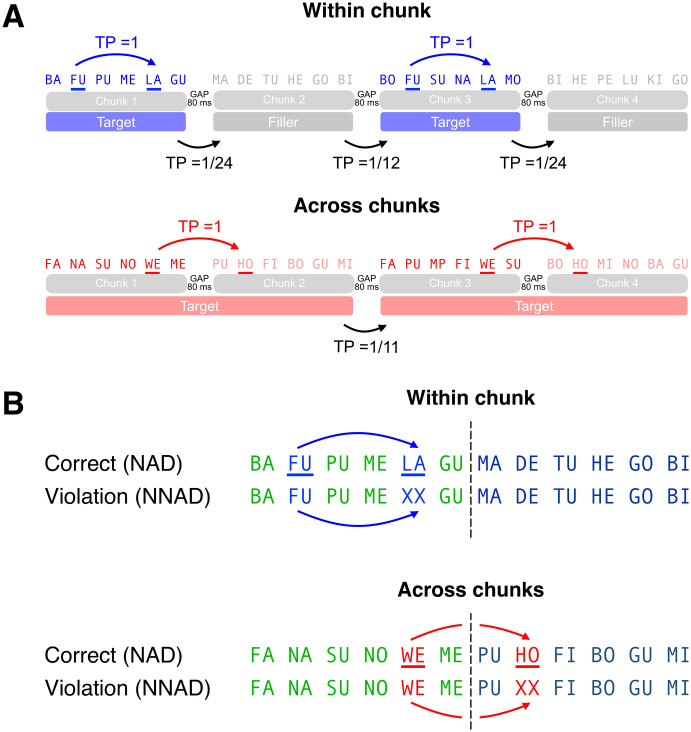
Experimental conditions. (A) Participants listened to both within-chunk and across-chunks in the learning phase. Target chunks in the within-chunk condition were intervened with the filler chunks to have the same amount of NADs as the across-chunk condition. The transitional probability between dependency is 1.; (B) Participants listened to both correct (NAD) and violation (NNAD) for both within-chunk and across-chunk conditions in the test phase.

But if we sample and process one chunk at a time, how can we link words that belong to separate chunks [40]—or can we? In the following, we present EEG results that show the chunk boundaries appear to block NAD processing and possibly learning. We designed an AG, composed of syllable sequences with equal duration. This allowed us to employ the frequency-tagging paradigm to assess whether people sample continuous speech into the desired size of units. Participants learned 6-syllable chunks from sequences, based on transitional probabilities and an additional short pause and the chunk boundary. Syllable streams either contain the NADs within a 6-syllable chunk or across two chunks (see Fig 2). Spectral analyses of EEG indeed revealed a spectral peak at the chunk rate, suggesting that people are able to learn the chunks. Crucially, smaller electrophysiological responses were observed in NADs that traversed chunk boundaries, compared to the NADs within a chunk. This suggests that NADs across chunk boundaries are harder to process than NADs within a chunk. Our findings can help to bridge the gap between incremental processing of acoustic signals, such as speech; moreover, they strengthen previously proposed links between periodic neurobiological activity and the chunking of speech.

## Materials and methods

**Fig 3:**
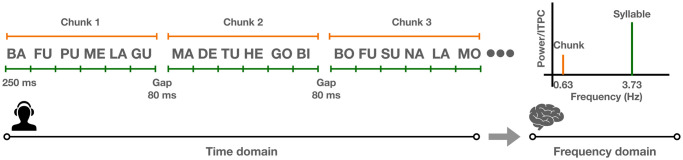
Frequency-tagging paradigm. Participants listened to a sequence of syllables. In the time domain (left), each syllable lasts 250 ms; each chunk consists of 6 syllables. An 80-ms silence was inserted between chunks. In the frequency domain (right), this leads to an according frequency of syllable occurrence of 3.73 Hz, while 6-syllable chunks occurred with a frequency of 0.63 Hz. When participants’ brains track both syllables and chunks, we expect to observe peaks in the EEG power / ITPC (= inter-trial phase coherence) spectra at both syllable and chunk rates.

**Fig 4:**
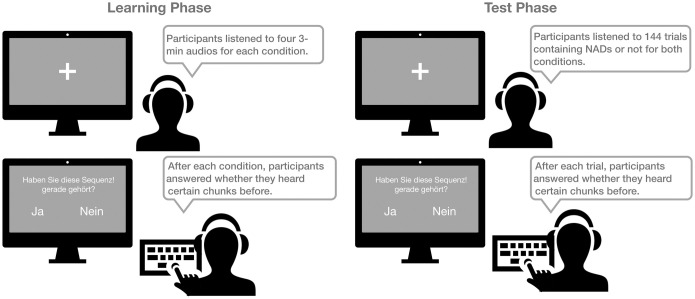
Experimental procedure. The whole experiment includes two phases: the learning phase and the test phase. During the learning phase, participants listen to four 3-minute audios for each condition and then answer 40 questions. During the test phase, participants answer a question after each trial.

To test the hypothesis of whether an NAD across chunks would be harder to process, the current study applies an AG learning paradigm combined with frequency tagging (see Fig 3). By using frequency tagging, we can see whether participants acquire chunks during the learning phase. Thus the learning phase serves two purposes: first, participants learn NADs within and across chunks; second, participants learn six syllables as a chunk. If participants are able to learn six syllables as a chunk, a peak at the chunk frequency should be observed. Then during the test phase, participants listen to trials with either correct NADs or incorrect NADs in both within-chunk and across-chunk conditions. If NAD processing is indeed harder across chunk boundaries, we expect the difference of amplitude from correct and incorrect elements that complete NADs would be smaller in the across-chunk condition. The overall experimental procedure is demonstrated in Fig 4.

### Participants

Thirty-seven German native speakers (18 females, 19 males) between the ages of 18 and 34 (mean = 24.5) participated in the experiment (recruitment period: 01/03/2022-31/07/2022). They were all right-handed and had normal hearing. They self-reported that they did not have any neurological disorders. They gave written informed consent before participation and were reimbursed for their time (9 Euros per hour). Data from four participants were excluded from the analysis due to technical recording issues and two were removed due to poor accuracy in the learning phase (< 50% accurate). Thus, data from 31 participants (16 female, 15 males) were included in the final analysis. The study was approved by the local ethics committee of the University of Leipzig (file 060/17-ek).

### Materials

German syllables (see Tables 1 and 2) were recorded individually as isochronous speech from Google Cloud Text-to-Speech (Male, de-DE-Wavenet-B). Complete stimuli can be found here: https://github.com/chiawenl/NAD-exp. Two native German speakers confirmed that syllables sounded naturally. Each syllable was adjusted to a duration of 240 ms and a 10-ms silence was appended to each syllable using the Praat vocal toolkit [41] in Praat [42] and customized Python scripts. Thus, each syllable lasts 250 ms. Six syllables were concatenated together to form a chunk. After each chunk, an extra 80-ms silence was appended to indicate a chunk boundary. These boundary silences were included to ensure that participants would succeed in learning the 6-syllabic chunks. It has been shown that prosodic cues aid both the formation of an NAD and the sampling of chunks [43, 44]. Critically, this intentional redundancy of transitional probability and pause duration would not affect the interpretability of any differences in NAD processing hypothesized to surface at the second element of the NAD (see Fig 2).

**Table 1:** Syllables for target and filler chunks.

Target syllable		Filler syllable	
1	BA	1	BI
2	BO	2	BU
3	FA	3	DE
4	FI	4	GO
5	GU	5	HE
6	ME	6	KI
7	MI	7	LE
8	MO	8	LI
9	NA	9	LU
10	NO	10	MA
11	PU	11	PE
12	SU	12	TU

**Table 2:** Syllables for dependency pairs.

DEPENDENCY PAIRS		
1	FU	LA
2	TA	PI
3	KE	MÖ
4	WE	HO

Twenty-four target chunks for each condition (12 for NAD and 12 for violation) in one list were created for each condition. To have equal length for both within-chunk and across-chunk conditions, 24 filler chunks constructed from filler syllables (Table 1) were created and inserted between target chunks in the within-condition. The same 12 target syllables and 12 filler syllables were used across participants. Four lists were created; each list includes two dependency pairs. One participant learned one dependency pair in the within-chunk condition and the other dependency pair in the across-chunk condition. Four dependency pairs (Table 2) were balanced across within-chunk/across-chunk conditions. Thus, each syllable in the pairs appears in the second and fifth positions equally.

For the learning phase, two experimental conditions were created—within-chunk and across-chunk. An example stimulus is shown in Fig 2. As for the test phase, four conditions were made—whether the second element of a dependency pair forms the dependency in both within-chunk and across-chunk conditions. The elements of a dependency pair were situated in the second and the fifth positions for the within-chunk condition while they were situated in the fifth position of the first chunk and the second position of the next chunk for the across-chunk condition. This positioning served to address the difficulty of ruling out the possibility that participants of prior studies were merely sensitive to detecting the edge of a chunk, rather than segmenting continuous stream by the formation of NADs [21, 45]: In some prior studies, the second element of the NAD co-occurred with the (prosodic) chunk boundary. Endres and Mehler [21] directly test the edge difference between strings (AXYZB vs. XAYBZ). They found that participants are indeed sensitive to the NADs that occurred at the boundary, compared to the medial positions. The remaining syllables were distributed evenly from the 12 target syllables for each condition.

Four 3-minute audios were created for both conditions for the learning phase. The within-chunk condition included 72 target chunks and 72 filler chunks per audio while the across-chunk condition included 144 chunks that contain a dependency pair across two chunks. Seventy-two filler chunks were inserted between target chunks in the within-chunk condition to balance the amount of NADs for both conditions.

For the test phase, 144 trials that include NADs or violations (NNADs) for both conditions were created. To avoid additional learning, trials with either correct NADs or violations for both conditions were randomly distributed during the test phase. Each list included 36 NAD trials and 36 violated trials for both conditions. Four target chunks that either include correct NADs or violations were included in one trial. Hence, 4 target chunks and 4 filler chunks were included in the within-chunk condition while 4 target-across-chunk were included in the across-chunk condition. After each trial, a comprehension question about whether a specific chunk in the trial they just heard was assigned. The answers were balanced.

### Procedure

Participants sat comfortably in front of a computer screen in a quiet room. Stimuli were presented using Presentation (Neurobehavioral Systems, Inc., Albany, US). Before the main session, participants were fitted with an electrode cap. Electrolyte gel was applied to minimize impedance below 10 kOhms. The setup took approximately 30 minutes.

The main session included two phases—the learning phase and the test phase (see Fig 4). Participants were instructed to listen to the audio carefully and avoid unnecessary body movement and frequent eye blinking during recording. During the learning phase, participants listened to four 3-minute audios in one condition and answered 40 questions about whether they just heard a specific chunk in the previous audios. Then, participants continued the other three 3-minute audios and 40 questions for the other condition. The order of conditions was counterbalanced. The learning phase took roughly 30 minutes to finish.

During the test phase, participants listened to 144 trials (in six blocks) that included either the corrected NAD or the violations in both conditions. Participants could take a short break after each block. Before the main test, participants had four practice trials to become familiar with the task. After each trial, participants had to answer whether they heard a specific chunk in each trial. The test phase took roughly 40 minutes to finish. After the test phase, participants removed the cap and a debrief of the goal of the experiment was given.

### EEG recording and data analysis

EEG data were recorded at 500 Hz from 63 Ag/AgCl electrodes mounted in an elastic cap (ANT Neuro GmbH, Berlin, DE) with online reference to the left mastoid (A1). Horizontal and vertical eye movements were monitored by the bipolar electrodes placed above and below the right eye and the electrodes placed on the outer canthi. An electrode on the stratum served as the ground.

EEG pre-processing was done by applying the modified Harvard Automated Pre-processing Pipeline [46] with a combination of EEGLAB [47] and FieldTrip [48] functions executed in Matlab (The MathWorks, Inc., Natick, US). Line noise was removed by applying Zapline-plus [49]. Then data were re-referenced offline to the average of the left and the right mastoid electrodes. Bad channels were recognized by the normed joint probability of the average log power and rejected if they were above the threshold of 3 SD (mean number of removed channels = 4.13, SD = 2.62). A high-pass filter of 0.1 was applied (FIR, Hamming windowed, reversed filtering), and then the data were re-referenced to the common average of all electrodes excluding the channels that were marked as bad (see also [50]). To obtain optimal decomposition from independent component analysis (ICA, [51]), a wavelet-enhanced independent component analysis (W-ICA, [46, 52]) before applying ICA to remove large artifacts. ADJUST [53] was applied to detect artifact components based on a set of temporal and spatial features of each component (mean number of removed components = 15.1, SD = 7.92). Data in the learning phase were epoched to 12.64 seconds, resulting in 8 chunks in one trial. Thus 72 trials were analyzed for each condition. For the test phase, the elements of dependency pairs were epoched from 100 ms pre-stimulus to 300 ms post-stimulus and then baseline-corrected. After epoching, FASTER [54] was applied to each channel per epoch to detect artifacts automatically. Channels were spline interpolated if it is contaminated within each epoch. Then channels that were recognized as bad previously were also interpolated by using surface spline interpolation [55].

As the goal of the spectral analysis during the learning phase was to see whether participants were able to derive 6-syllable chunks, we collapsed and analyzed neuronal synchrony across all trials in both conditions together. The target frequency for the syllable rate is around 3.73 Hz and for the chunk rate is 0.63 Hz. Neuronal synchrony was assessed from Evoked Power (EP) and Inter-trial Phase Coherence (ITPC), following the algorithm defined by [56]. EP (Eq (1)) reflects the power of EEG responses synchronized with speech stimuli in both phase and time. *X*_*n*_(*f*) is the summation of complex-value Fourier coefficient of trials derived from the Discrete Fourier Transform. EP is obtained from *X*_*n*_(*f*) averaged over the total number of trials *N*. We computed EP from 0.1 to 10 Hz in increments of 0.079 Hz. The 1/f noise in the power spectrum was normalized by dividing the value at the target frequency from the average of neighboring values within ±0.5 Hz via the Eq (2) adopted from [56], where *w* represents the neighboring frequency around the target frequency *f*. There are many ways to normalize power (e.g. Irregular-resampling auto-spectral analysis (IRASA, [57]), fitting oscillations and one over *f* (FOOOF, [58]). The approach for normalization applied here has been shown that yield similar results by applying other normalization (see also [59]). ITPC reflects the phase consistency across trials. ITPC (Eq (3)) is obtained by averaging over the total number of trials *N* from the square root of summation of cosine and sine values of phase angles *θ*_*n*_ of each complex-value Fourier coefficient. For statistical analysis, normalized EP and ITPC of target frequencies (chunk and syllable rate) were compared with the neighboring 4 frequency bins around the target frequencies.

$$\begin{array}{c} \text{EP}\left(f\right)=\frac{|\sum_{n}X_{n}\left(f\right){|}^{2}}{N} \end{array}$$ (1)

$$\begin{array}{c} \text{EPn}\left(f\right)=\frac{E(f)}{\sum_{w}E\left(w\right)},|w-f|<0.5\;\text{Hz},w\neq f \end{array}$$ (2)

$$\begin{array}{c} \text{ITPC}\left(f\right)=\frac{\sqrt{{\left(\sum_{n}\left(cos\theta_{n}\right)\right)}^{2}+{\left(\sum_{n}\left(sin\theta_{n}\right)\right)}^{2}}}{N} \end{array}$$ (3)

To examine whether the NAD across chunks is harder than the NAD within a chunk in the test phase, we analyzed the magnitude of the second element that forms or violates dependency in the event-related component (ERP) with time-locked to the syllable onset. A low-pass filter at 25 Hz (IIR, two-pass filtering, and Hamming windowed, default in Fieldtrip) was applied to the epoched data before group analysis. A non-parametric permutation test [60] was conducted to correct multiple comparisons across all electrodes time-locked to the second element, which forms a correct or incorrect dependency. For each condition, the permutation test was conducted by following these steps: (i) Dependent samples T-statistics were conducted at each time point and electrode, (ii) tests with *p* < 0.05 were clustered based on spatial-temporal adjacency and their T-statistics were summed by using weighted cluster mass, a method that takes cluster size and intensity into account [61], (iii) Steps (i) and (ii) were repeated 10,000 times by randomly permuting the conditions for each subject, and (iv) clusters with summed statistics that surpassed at least 95% from the permutation test were kept as “statistically significant”.

## Results

### Chunks are learned from syllable streams

**Fig 5:**
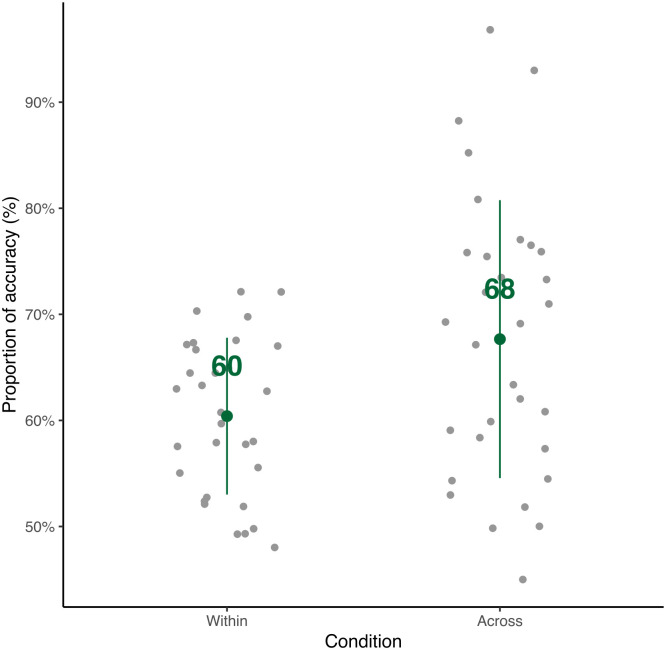
Behavioral results in the learning phase. The overall accuracy is 64%. The gray dot indicates the score for each participant. The green dot indicates the mean for each condition and the green line indicates the standard deviation.

**Fig 6:**
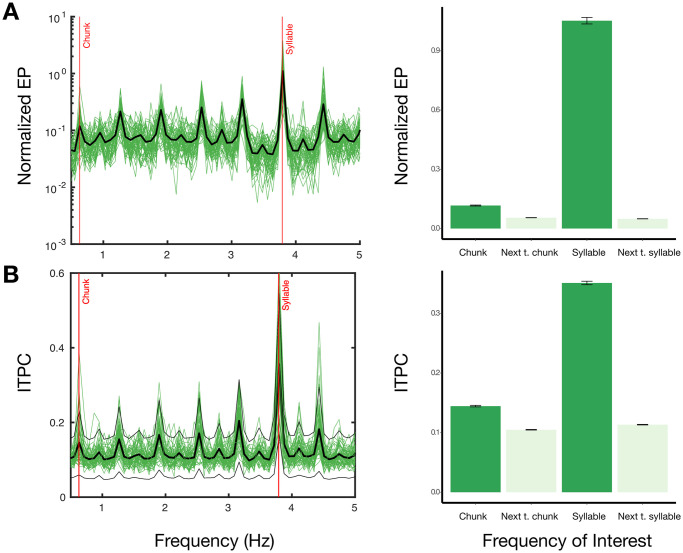
Results of normalized EP and ITPC in the learning phase. (A) Results of normalized EP: Peak at the syllable and chunk rates are marked as red in the power spectrum (left). Green lines indicate individual subjects. The black line indicates the average across subjects. The right bar graph shows the peak values from the chunk and syllable rate (deep green) and the average from the neighboring two frequencies around the chunk and syllable rate (light green). (B) Results of ITPC.

The overall accuracy of the comprehension questions is 64% correct in the learning phase, indicating chunk learning. The accuracy for each condition is shown in Fig 5. The Paired *t*-test testing the difference between the two conditions (mean of within-chunk: 60%; mean of across-chunk = 68%) shows that the accuracy in the across-chunk condition is significantly higher than the accuracy in the within-chunk condition (*t*(30) = -2.9, *p* = 0.007). The higher accuracy of the across-chunk condition might be due to the fillers inserted in the within-chunk condition. Since more novel syllables needed to be learned, hence increased noises and lowered accuracy in the within-chunk condition. To assess neural synchrony, normalized EP and ITPC were computed. Fig 6A shows the power spectrum during the learning phase. We used *lme4* [62] in R [63] to fit a linear mixed effect model (estimated using REML and nloptwrap) to test normalized EP and ITPC of target frequencies and neighboring frequencies (formula: EPn ∼ type; ITPC ∼ type). The models include Subject as a random effect (formula: 1|Subject). For normalized EP, the model’s intercept, corresponding to neighboring frequencies around the chunk rate, is at 0.05 (*t*(15122) = 1.78, *p* = 0.076). Within this model, the peak of averaged neighboring frequencies around syllable rate is statistically non-significant (beta = -0.004, *t*(15122) = -0.33, *p* = 0.74). The peak of chunk frequency is statistically significant (beta = 0.07, *t*(15122) = 5.56, *p* < .001). The peak of syllable frequency is also statistically significant (beta = 1.05, *t*(15122) = 87.82, *p* < .001). The post-hoc tests using Kenward-Roger methods in *lmerTest* [64] further confirm that there is a significant difference between the normalized EP of the chunk rate and the normalized EP of the neighboring frequencies around the chunk rate (*t*(15094) = -5.56, *p* < 001). There is also a significant difference between the normalized EP of the syllable rate and the normalized EP of the neighboring frequencies around the syllable rate (*t*(15094) = -88.15, *p* < .001). There is no significant difference between the normalized EP of the neighboring frequencies around the chunk and the syllable rate (*t*(15094) = 0.33, *p* = 0.74).

Fig 6B shows the results of ITPC. The model’s intercept, corresponding to frequencies around the chunk rate, is at 0.10 (*t*(15122) = 17.33, *p* < .001). Within this model, we found that the ITPC of the chunk rate is statistically significant (beta = 0.04, *t*(15122) = 17.98, *p* < .001). The ITPC of the syllable rate is also statistically significant (beta = 0.25, *t*(15122) = 108.2, *p* < .001). The ITPC of neighboring frequencies around the syllable rate is statistically significant (beta = 0.01, *t*(15122) = 4.35, *p* < .001). The post-hoc tests show that there is a significant difference between the ITPC of the chunk rate and the ITPC of the neighboring frequencies around the chunk rate (*t*(15094) = -17.98, *p* < .001). A significant difference was also found in the comparison between the ITPC of the syllable rate and the ITPC of the neighboring frequencies around the syllable rate (*t*(15094) = -103.86, *p* < .001).

Results from both EP and ITPC have shown that the peaks at the chunk and the syllable rate can be observed. The results suggest that participants’ brains identify and represent six syllables. In line with previous work [43], this indicates that participants are able to learn chunks based on transitional probabilities and additional short pauses.

### Processing of within– but not across-chunk NADs

**Fig 7:**
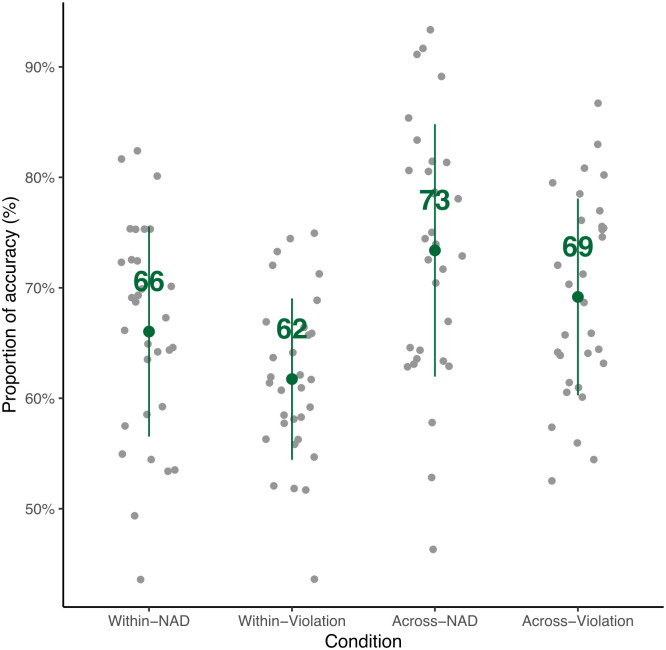
Behavioral results in the test phase. The overall accuracy is 68%. The gray dot indicates the score for each participant. The green dot indicates the mean for each condition and the green line indicates the standard deviation.

The overall accuracy of the behavioral responses is 68% correct in the test phase, indicating continued memory of the learned chunks. The accuracy across different conditions (Within-chunk vs. Across-chunk; NAD vs. Violation) is shown in Fig 7. We fitted a linear mixed model (estimated using REML and nloptwrap optimizer) to test the accuracy with the two conditions (within-chunk vs. across-chunk) and whether the dependency is correct (formula: accuracy ∼ within/across * dependency). The model included Subject as a random effect (formula: ∼1 | Subject). The effect of within/across is statistically significant (beta = -0.07, *t*(118) = -3.55, *p* < .001). The dependency effect is statistically significant (beta = -0.04, *t*(118) = -2.04, *p* = 0.044). The two main effects have no significant interaction (beta = -0.009, *t*(118) = -0.03, *p* = 0.976). Similar to the behavioral results in the learning phase, accuracy in the across-chunk condition is higher than the one in the within-chunk condition. Within each condition, the accuracy of the correct dependency is significantly higher than the accuracy of the violation (Post-hoc Within-NAD vs. Within-violation: *t*(90) = 2.08, *p* < .05; Across-NAD vs. Across-violation: *t*(90) = 2.04, *p* < .05).

**Fig 8:**
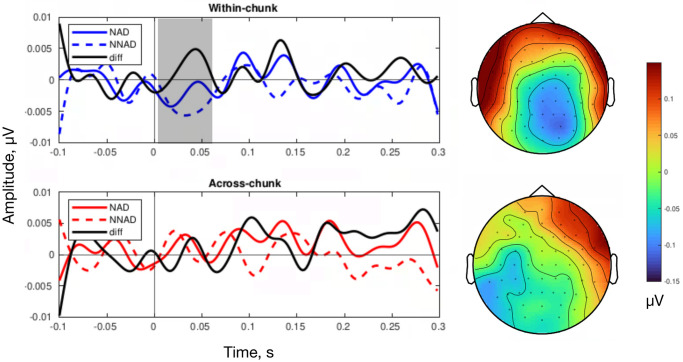
ERP results in the test phase. ERPs for the second element of correct NAD and violation (NNAD) for each condition. There is a statistical difference between the NAD and the NNAD in the within-chunk condition (the gray area), but not in the across-chunk condition. The black line indicates the difference between the correct and incorrect elements. Topographies show the difference between the NAD and the NNAD between 0-50 ms for each condition.

Event-related potentials (ERP) were computed to assess whether NADs across chunks are harder to process. ERPs at the second elements that either complete an NAD (NAD) or violate it (NNAD) were compared within each condition. Fig 8 illustrates the results. The difference between NAD and NNAD syllables is significantly larger around 0-0.05 seconds in the within-chunk condition (*p* = 0.0017) while there is no significant difference in the NAD and violation in the across-chunk condition (*p* = 0.08). The difference found in the within-chunk condition but not in the across-chunk condition suggests that NADs across chunks are indeed harder to process.

## Discussion

Our results dissociate and link the active segmentation of continuous speech into multi-word memory chunks and the incremental construction of compositional meaning through NADs. This may help to reconcile disparate aspects of human language comprehension: On the one hand, in the face of memory limitations, the human brain needs to sample speech in short chunks; on the other hand, our brains link words incrementally, often requiring the formation of NADs. The findings in the current study serve as the initial electrophysiological evidence that NAD processing is restricted to the current memory chunk, which had been previously only shown by computational modeling [30] and behavioral studies [20, 25]. Frequency-tagging results converge on prior work that suggests that the brain actively segments continuous speech into discrete chunks with the help of low-frequency activity [37–39]. ERP results indicate that NADs are harder to process once the dependencies cross the chunk boundary. Critically, our results overcome ambiguities in prior research, which mostly defined chunk boundaries by NADs themselves, making it difficult to dissociate the processing of segmentation and the formation of dependencies.

The current results further indicate that comprehension involves the sampling of chunks in a shallow manner, in line with previous psycholinguistic work [65–67] and neuroscientific studies [68]. In the background of building dependencies amongst incoming words incrementally in real-time, speech is sampled superficially in coarse second-long chunks; here, subjects were able to learn these based on both statistical and prosodic cues. Structure building, as operationalized here through the statistical co-occurrence of the first and second elements of NADs, seems incremental, yet constrained by the boundaries of memory units. This is consistent with the chunk-and-pass model of language processing, which adopts an incremental approach and requires people to integrate information as quickly as possible [40]. Our results are aligned with earlier processing models such as the sausage machine [35] and provide a link with incremental approaches [69].

**Fig 9:**
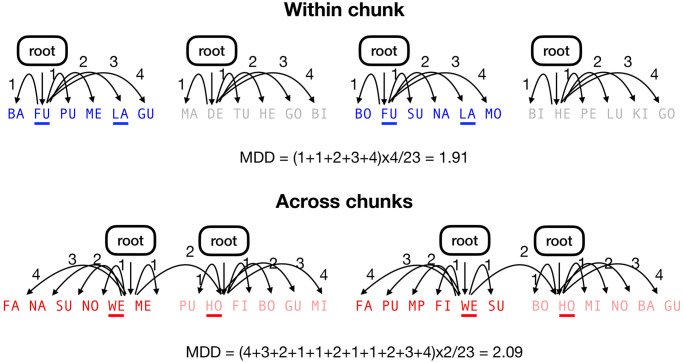
An example of MDDs in both conditions. The MDD is higher in the across-chunk condition than the MDD in the within-chunk condition.

The spectral peak observed in the learning phase provides supporting evidence that neural activity in the delta band underlies chunking, consistent with recent findings [37–39]. This might entail that the cognitive units of language comprehension are clusters held together by local transitional probabilities. The current memory chunk may thereby allow for establishing all dependencies required for understanding the current chunk. An exploratory analysis, using the metric of mean dependency distance (MDD; see Eq (4)), following corpus simulations from [70], suggests that this would indeed be an effective manner of processing. MDD quantifies syntactic complexity by measuring the distance between each word and chunk. In the Eq (4), *n* refers to the number of element/word and *k* refers to the number of chunks; *cdd* refers to the distance within the *i*th chunk and *ldd* refers to the distance between chunks. Indeed, MDD is higher for across-chunk dependencies, an example demonstrated in Fig 9. In line with the rarity of crossing dependencies found in natural languages, our results show that syntactic complexity may be reduced due to the proper local arrangement of units to be linked (in our AG: syllables; in natural language: words/morphemes). This is consistent with the chunk-and-pass model of language processing [40], evidence for dependency length minimization in the world’s languages [27, 29], and the dualism of segmentation and parsing in classical psycholinguistic frameworks of sentence processing [35, 71]. Yet, our findings suggest that NAD length is not only limited by memory constraints and flexible otherwise— apparently, our cognitive flexibility to form NADs is also constrained by the boundaries of the memory units segmented from speech. The rarity of crossing dependencies may result from the duality of language [70]. The encoding of sound structure and the composition of meaningful words or morphemes happen rapidly, incrementally, and concurrently; thus, information can be integrated locally and the possibility of crossing dependencies may be reduced. Hence, our memory capacity would not be overloaded due to this multi-level parallel processing and thus achieve successful language comprehension and production. Beyond the corpus simulation results in [70], we further show that the reduction of syntactic complexity may be observed on the neural level. Future directions addressing how different boundaries (i.e., syntactic boundary vs. prosodic boundary) may elicit similar interference and how different types of syntactic dependencies (e.g. nested dependencies in German and crossed dependencies in Dutch, [72]) are disrupted will be key to further disentangling how chunk boundaries interfere with the formation of NADs.

$$\begin{array}{c} MDD=\frac{1}{n-1}\sum_{n=1}^{k}\left(|cdd_{i}|+|ldd_{i}|\right) \end{array}$$ (4)

It must be debated whether our findings can be taken to fully generalize to natural language processing. As criticized by previous research, most AGs lack syntactic word categories (parts of speech) and the hierarchical rules that many researchers assume to be found in natural language; both may limit the generalization to natural language processing [73]. However, previous studies have also shown that the transitional probabilities that define AGs capture at least some aspects of the cognitive form of linguistic knowledge. For instance, phonotactic statistical knowledge of a language stored in our long-term memory can indeed influence how we recall non-words [74–76]. That is, non-words are better recalled when the transitional probabilities of non-words are consistent with the pattern in natural language (see [77] for further discussion on memory). Therefore, we do suggest that the AG paradigm here captures the dualism of chunking and NADs also found in natural language (processing) reasonably well. We should also note that, for natural languages, we are still able to link words across chunks. Yet, it remains to be tested whether the current experimental design, transposed to natural language, will similarly show that this ability is less articulate than NAD processing within chunks. For future research, as suggested by [73], a combination of an AG with pseudo-words could be used. Results may be more comparable with natural language processing.

In addition to transitional probabilities, our experimental setup ensured the formation of chunks through a short pause and the chunk boundary. Pauses certainly support chunking. For instance, low-frequency periodicity was observed in speech sequences defined by intonation units, defined, amongst other factors, by pauses [78, 79]. Neural responses track both overt and covert prosodic boundaries [80] and chunk boundaries defined by intonation units [81]. Neural activities in the delta band may be affected by both prosodic information and syntactic structure simultaneously and the processes of these two kinds of information are overlapped strongly [82, 83]. Future research is required to see how prosody and syntax play a role in defining an optimal memory chunk formed by local dependencies. In any case, the combined marking of chunk boundaries by transitional probabilities and a short pause in the current study leaves the NAD-blocking effect untouched.

Our ERP results during the test phase reveal an unexpectedly early effect, inconsistent with the previous findings that would let us expect a modulation of the N100 component, indexing form-based processing of words during the first 80-120 ms [84–86]. However, this early effect is plausible for linguistic processing. For example, Herrmann and colleagues [87] found a greater activation between 40-80 ms for the incorrect phrases during an MEG recording of an auditory oddball paradigm for syntactic stimuli (see also [88]). Another study also shows an early effect around 20-100 ms for the violation of grammatical categories during sentence listening [89]. As mentioned by Herrmann and colleagues [87], this earlier effect is possibly associated with the P50 component, which has been associated with preferential attention to sensory inputs and general auditory arousal [90], stimulus onset perception [91], and phonemic encoding [92]. In line with the assumption of the relationship between the P50 and the early grammatical effect in [87], it seems plausible that the dependency processing can be modulated by preferential attention to sensory inputs in our finding. Critically, the current NADs could be processed on their acoustic/auditory form alone (i.e., subjects needed to learn syllable form), which overlaps in time with the current result [93–97]. Still, more future research is needed to investigate how this early effect is associated with dependency processing and how perception interacts with comprehension of higher-level information at the very early stages of language comprehension.

Future work should also extend the current findings to language development. It has been shown that infants store large chunks in early development [98, 99]. Infants are initially sensitive to slow prosodic information, which marks phrase or clause boundaries—chunk boundaries. Slowness facilitates the neural tracking of prosody in infants [100, 101]. In addition, infants 8 months of age track statistical regularities in speech and exploit transitional probability to segment continuous speech [14]. The detection of NADs can also be observed at a very young age. A study has shown that 3-month-old infants can detect the violation of AXB, accompanied by their auditory processing capacities [102]. Other studies have also shown that 8-month-old infants can learn the NADs from an AG [103, 104]. At roughly 16 months, children learning English can recognize the grammatical dependency between auxiliary and inflectional morphology [24] (e.g. *Everybody is always baking bread.*). Similar evidence was also observed in children across different languages (German: [105], French: [25, 106], Dutch: [107]). Remarkably, a study has shown that 17-month-old infants can track NADs even when two elements are across different phonological units [25]. This indeed leaves us asking how infants integrate phonological and syntactic information during oscillatory chunk sampling. Infants start from sampling in larger chunks and then recognize the complex structures such as NADs in real language late. How children deal with the coarse units and the NAD processing remains unknown. If children sample in larger units and those units are able to include NADs involving longer distances, would the distance of NAD show a reduced effect in children, compared to adults? In addition, children’s strategies for segmentation may change across development; that is, children may rely on acoustic information (e.g., stress) initially and later adapt their strategy to statistical cues to segment continuous speech [17]. How this dynamic adaption to different strategies for speech segmentation across development interacts with the processing and learning of NADs remains a major puzzle for language acquisition research. These possible directions would be fruitful for future research and could add a valuable dimension to early language development.

## Conclusion

We show that learners segment continuous sequences into chunks, possibly with the help of delta-band oscillations. Cognitive chunk boundaries then block NAD processing. Chunk-wise sampling and NAD processing go hand in hand, with NAD processing operating within the current memory chunk. Results link the segmentation of speech by our memory-limited brains and the formation of NADs that are the combinatorial basis of human language. For further application of the current research, this can be possibly applied to language teaching. For example, students might acquire number/gender agreement or filler-gap dependency more easily if they can identify the plausible multi-word chunks in a language. Teachers can start teaching NADs within small plausible multi-word chunks when teaching a foreign language. After students gain sensitivity to the NADs within a chunk, they can gradually extend the chunk size and then form NADs across chunks. In future directions, it will be worth investigating how different types of chunk boundaries, also in natural language, (i.e., syntactic boundary vs. prosodic boundary) may drive similar types of interference and whether different types of NADs could be disrupted equally by chunk boundaries. Additionally, as children acquire larger chunks in early language development, examining how different segmentation strategies interact with the formation of NADs in language acquisition would be promising.
